# Optimized feature selection and zero-parameter channel attention BiLSTM for RPL-attack classification in IoT networks

**DOI:** 10.1371/journal.pone.0350844

**Published:** 2026-06-18

**Authors:** Sudha Rani Unnam, Kareemulla Shaik

**Affiliations:** School of Computer Science and Engineering, VIT-A.P. University, Amaravati, Andra Pradesh, India; KTH Royal Institute of Technology: Kungliga Tekniska Hogskolan, SWEDEN

## Abstract

The increasing adoption of Internet of Things (IoT) devices introduces significant security challenges, particularly in networks based on the Routing Protocol for Low-Power and Lossy Networks (RPL), where resource constraints limit the effectiveness of conventional security solutions. This work presents an optimized deep learning framework for detecting and classifying RPL-based routing attacks. The framework integrates Chaotic Pied Kingfisher Optimization (Ch-PKO) for feature selection with a Zero-parameter Channel Attention Bidirectional Long Short-Term Memory (ZCAtt-BiLSTM) model for classification. Data preprocessing includes cleaning, one-hot encoding, and Pareto scaling to improve data quality and learning stability. The approach is evaluated on the IoT-RPL dataset, covering Blackhole, Flooding, DODAG Version Number, and Decreased Rank attacks. Experimental results show strong performance, achieving 99.305% accuracy, 98.57% F1-score, 0.61% false discovery rate, and 97.949% MCC. Comparative analysis indicates improved performance over baseline models. However, the evaluation is based on simulated data, and the training process introduces additional computational cost, which may affect real-time deployment.

## Introduction

The Internet of Things (IoT) represented a critical domain within information technology, interlinking smart devices to enable seamless communication and data sharing across diverse environments. In recent decades, the number of IoT-enabled devices has grown exponentially, and industry forecasts predict that by 2025 the global IoT ecosystem will surpass 100 billion connected devices [1,2]. This rapid growth inevitably gave rise to large-scale, highly complex network infrastructures that processed vast amounts of data in real time. Alongside these benefits, IoT systems faced mounting security concerns that threatened their reliability and trustworthiness. Among the various routing protocols designed for IoT environments, the Routing Protocol for Low-Power and Lossy Networks (RPL), introduced by the Internet Engineering Task Force (IETF), emerged as the most widely used [3,4]. RPL was designed to optimize routing in resource-constrained networks, offering flexibility across diverse topologies and energy-efficient communication. Its adaptability and low computational requirements made it indispensable for applications such as smart homes, industrial automation, and environmental monitoring, where reliable and continuous data transmission was essential [5,6]. Despite these advantages, RPL’s open architecture and lightweight design rendered it highly susceptible to attacks, placing IoT networks at significant risk.

The core mechanisms of RPL exposed multiple vulnerabilities that adversaries exploited to launch severe routing-based attacks. In Blackhole attacks, malicious nodes advertised false optimal routes and subsequently dropped all intercepted packets, resulting in substantial data loss [7,8]. Flooding attacks exploited the lack of strong authentication and resource constraints of IoT nodes by injecting excessive control or data packets, rapidly depleting energy and bandwidth while causing network congestion [9]. DODAG Version Number attacks manipulated control messages by broadcasting fraudulent version numbers, forcing unnecessary topology reconstructions that degraded performance and stability [10,11]. Similarly, Decreased Rank attacks falsified rank values to mislead nodes into adopting compromised routes, disrupting network paths and undermining overall reliability. Collectively, these attacks compromised data confidentiality, integrity, and availability, making cyberattack detection in RPL-based IoT networks a critical research challenge [12,13].

In response to these threats, a wide range of attack detection techniques was investigated for IoT systems. ML approaches, in particular, showed considerable promise by analyzing large volumes of network traffic to uncover both known and previously unseen attack patterns [14,15]. By learning complex behavioural signatures from IoT data, machine learning-based systems enhanced detection accuracy and extended the scope of protection against evolving cyber threats. However, despite these advances, existing detection methods still suffer from notable shortcomings [16,17]. Many of the proposed solutions concentrated narrowly on defending against a single type of attack, which limited their applicability when multiple attack vectors occurred simultaneously [18]. Moreover, these approaches frequently relied on computationally intensive algorithms that were unsuitable for resource-constrained IoT devices, making them impractical in real-world deployments. Another critical limitation was that most existing systems struggled to achieve real-time detection while maintaining low computational overhead, an essential requirement for IoT security. These challenges underscore the inadequacy of current solutions in providing robust, scalable, and efficient cyberattack detection against the diverse and continuously evolving threats targeting RPL networks [19,20]. This article introduced a novel DL technique with an effective feature selection scheme for the classification of RPL-IoT attacks.

### Motivation

The increasing prevalence of network attacks targeting the RPL protocol in IoT networks created an urgent need for robust and efficient detection mechanisms. Conventional ML techniques, although widely explored for RPL-based cyberattack detection, suffered from several limitations when applied to the dynamic and resource-constrained nature of IoT environments. Many traditional classifiers relied heavily on manually engineered features, which made them sensitive to noise, redundant attributes, and irrelevant information present in large-scale IoT traffic. This often resulted in reduced detection accuracy, poor adaptability to new or sophisticated attack types, and higher false positive rates. Moreover, conventional ML models typically require significant preprocessing and handcrafted feature extraction, which not only increases computational complexity but also restricts scalability in real-time applications. These limitations made it challenging to effectively classify diverse attacks such as Blackhole, Flooding, DODAG Version Number, and Decreased Rank attacks. In contrast, deep learning techniques provided an effective solution by automatically learning hierarchical representations from raw or minimally processed data, thereby enhancing the ability to capture complex attack patterns and improving detection accuracy. However, DL models still faced performance degradation if trained on high-dimensional or redundant feature sets. To address this, the integration of feature selection techniques became essential, as they reduced dimensionality, eliminated irrelevant data, and highlighted the most informative features. This not only improved the efficiency and interpretability of DL models but also minimized computational overhead, making them more suitable for upcoming real-time attack detection in resource-constrained RPL-IoT networks.

***The foremost contributions of the proposed framework were encompassed as follows:***

• To have proposed an optimized deep learning framework that combined advanced preprocessing using Pareto scaling normalization, and Chaotic Pied Kingfisher Optimization (Ch-PKO) for effective feature selection, reducing dimensionality, and minimizing computation time.
• To have designed a Zero-parameter Channel Attention Bidirectional Long Short-Term Memory (ZCAtt-BiLSTM) model capable of accurately classifying multiple RPL-specific attacks, including Blackhole, Flooding, DODAG Version Number, and Decreased Rank attacks.
• To have validated the proposed approach through Python-based simulations and demonstrated the superiority of the proposed approach by comparing its results with existing state-of-the-art techniques, showing enhanced detection accuracy and reduced false positives.

The upcoming sections were prearranged as follows: Section 2 outlined the related work, Section 3 deliberated over the suggested approaches, Section 4 presented the results and discussion, and Section 5 represented the conclusion of the proposed framework.

### Related works: A brief review

**Fig 1:**
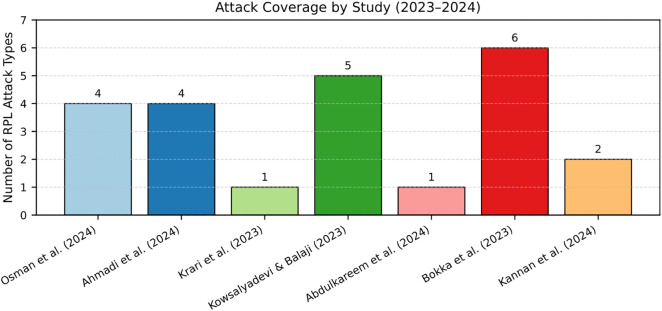
Attack coverage by recent RPL-based studies (2023–2024).

Among the several studies on cyberattack detection in RPL-IoT technology, several current works were deliberated in this section. Fig 1 illustrates the various studies and their respective attack coverage in RPL-based IoT networks.

In 2024, Osman et al. [21] introduced an innovative hybrid autoencoder–decision tree framework (HADTF) to detect four major types of RPL attacks, namely decreased rank, version number, DIS flooding, and blackhole attacks. The framework consisted of three primary components: enhanced feature extraction, feature selection, and an HADTF classifier. In the enhanced feature extraction stage, the most relevant features were derived from the raw network data. Subsequently, the feature selection process curated the optimal subset of features to reduce dimensionality and eliminate redundancy. Finally, the HADTF integrated the representation power of AE with the decision-making capability of decision trees, which led to improved accuracy and higher detection rates while minimizing both false positives and false negatives. The effectiveness of the HADTF was evaluated using a self-generated dataset. However, the data preprocessing and feature extraction required collecting detailed network traffic, which might introduce communication overhead in real deployments.

In 2024, Ahmadi et al. [22] elucidated an approach to address the susceptibility of the RPL protocol to various known routing attacks by introducing a trust-based identification framework. The expected behaviour of network nodes was estimated using a learning framework trained on historical routing patterns, leveraging recurrent neural networks (RNNs). This approach enabled the identification of attacks with notable accuracy and precision. However, its performance might vary under dynamic network conditions and with limited historical data, indicating potential constraints in real-time adaptability.

In 2023, Krari et al. [23] developed an innovative method for identifying the critical RPL VN attack within IoT networks by leveraging LSTM networks and DNNs. Through training the LSTM and DNN models on this dataset, complex behavioural patterns linked to the attack were captured to enhance detection accuracy. Despite its effectiveness, the approach might encounter challenges in handling real-time constraints or adapting to evolving attack strategies without frequent retraining.

In 2023, Kowsalyadevi and Balaji [24] encompassed the IoBTSec-RPL to recognize and classify numerous routing attacks in RPL-based networks. The framework followed four main stages: data preprocessing using min-max normalizer and imputation, feature selection via an enhanced pelican optimizer, class balancing with an auxiliary classifier gated adversarial network, and final classification using a combined LSTM and DBN. The model effectively captured complex attack patterns and demonstrated strong detection capabilities. However, scalability and real-time performance in large-scale dynamic IoT environments remained a challenge.

In 2024, Abdulkareem et al. [25] presented the BC with the VGG16 scheme to ensure data privacy and secure message transmission. Initially, network fabrication was carried out to optimally position the Mr. Fixit nodes and adapt to network dynamics using the Bettered Remora Algorithm (Be-Remo). Following this, the RPL topology was registered at the Dutiful Advisor (DA) by submitting relevant parameters, after which the DA issued secret keys to nodes via the Boosted CHACHA algorithm (B-CHACHA). The root node in the RPL then propagated DIO messages, allowing each node to choose its parent based on various optimal metrics through the Be-Remo mechanism. These requirements might limit deployment in resource-constrained IoT environments, and real-time responsiveness could be affected under high network load or frequent topological changes.

In 2023, Bokka et al. [26] emphasized the GRU-based DL model to detect security threats in RPL IoT networks. The dataset included traffic traces from both normal and attack scenarios, such as SH, BH, Sybil, Selective Forwarding, DIS flooding, and DIO suppression. The GRU model demonstrated strong detection accuracy across multiple attack types. Nonetheless, its performance under dynamic topologies and varying node mobility patterns was not fully explored.

In 2024, Kannan et al. [27] established the classification method using a combination of neural networks and genetic algorithms (NGCA). This NGCA effectively identified nodes responsible for RPL and other forms of attacks, thereby enhancing security within IoT networks. Experimental evaluation using the NSL-KDD dataset demonstrated that NGCA achieved higher detection accuracy compared to conventional classifiers. However, its performance might vary under different network conditions and datasets.

In 2025, Alemayew et al. [28] suggested a federated DL framework to identify and classify RPL attacks, including blackhole (BH), hello flooding (HF), and version number (VN). A multiclass dataset from IRAD environments, spanning 10–1000-node network sizes, was used for evaluation. Hybrid feature selection combined with Random Forest and XGBoost helped extract significant features. A CNN–GRU architecture captured both spatial and temporal characteristics of network traffic. Decentralized training was enabled through federated learning while keeping data local to clients. The model was compared with CNN-LSTM, LSTM, and GRU baselines. Results indicated improved detection performance across varying network scales. However, higher communication overhead and challenges due to non-uniform client data distribution limited efficiency.

In 2026, Nassrullah et al. [29] demonstrated the lightweight secure operating mode for the RPL protocol, termed LSM-RPL, to enhance network security. Hash-Based Message Authentication Code (HMAC) was employed to ensure source authentication and data integrity. Two categories of pre-configured keys were utilized for secure communication. A shared secret key was applied across all nodes to safeguard broadcast messages and block external attacks. A separate private key, maintained between individual nodes and the root, was used to secure unicast communication and mitigate internal threats. This dual-key mechanism strengthened both external and internal attack resistance. Simulation outcomes indicated improved resilience against common RPL attacks. However, key management complexity and potential scalability challenges in large networks remained notable limitations.

In 2025, Yang et al. [30] defined a routing attack dataset encompassing four RPL attack types, generated through simulations on the Cooja IoT platform. Feature extraction algorithms were implemented to derive 24 relevant attributes for the IDS model training. An efficient detection approach was designed to improve training speed and classification performance. A hybrid Pearson correlation was applied to identify the most informative features across different attack patterns. A GS-PSO (Grid Search–Particle Swarm Optimizer) strategy was utilized to optimize Random Forest hyperparameters. This combination enhanced detection accuracy while reducing computational complexity. The method demonstrated improved efficiency in identifying routing attacks. However, dependence on simulated data and potential performance variation in real-world deployments remained key limitations.

### Research gaps

Despite significant advancements in RPL attack detection and mitigation, several research gaps remain evident. Existing approaches primarily rely on simulated or self-generated datasets, limiting generalizability to real-world IoT deployments with dynamic and heterogeneous conditions. Many deep learning–based methods, including AE, LSTM, and GRU models, achieve high accuracy but incur substantial computational and communication overhead, making them less suitable for resource-constrained environments. Although feature selection and optimization techniques improve performance, they often depend on centralized training, raising concerns related to data privacy and scalability. Federated learning frameworks partially address privacy issues; however, challenges such as non-IID data distribution, communication cost, and client heterogeneity are insufficiently handled. Trust-based and cryptographic mechanisms enhance security but introduce additional complexity in key management and latency. Furthermore, limited attention has been given to real-time adaptability, evolving attack patterns, and lightweight model design. Scalability across large-scale IoT networks and robustness under varying topologies and mobility conditions also remain inadequately explored. These limitations highlight the need for efficient, scalable, and privacy-preserving detection frameworks capable of operating effectively in realistic and dynamic IoT environments.

## Methodology

**Fig 2:**
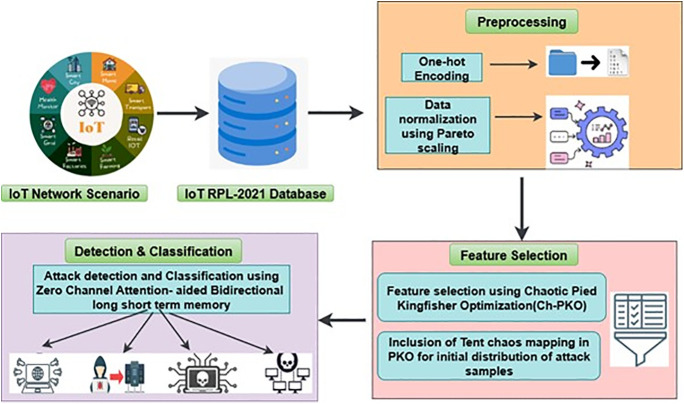
Workflow of the proposed method.

This section presents a structured description of the developed framework for detecting and classifying RPL-based attacks in IoT networks. The overall process consists of data acquisition, preprocessing, feature selection, deep learning-based classification, and performance evaluation. The complete workflow is illustrated in Fig 2.

Initially, the raw dataset from the publicly available source was collected. The raw data were then pre-processed by performing data cleaning, one-hot encoding, and Pareto-scaling-based data normalization. Following this, feature selection was performed by using Chaotic Pied Kingfisher Optimization (Ch-PKO) to optimally select the required attack features for minimizing computational overhead. Finally, the Zero-parameter channel attention Bidirectional Long Short-Term Memory (ZCAtt-BiLSTM) model was introduced to accurately classify various attacks like Blackhole Attack, Flooding Attack, DODAG Version Number Attack, and Decreased Rank Attacks. The proposed method was simulated via the Python platform, and various performance measures were examined and associated with other techniques.

### Data acquisition

An openly available IoT-RPL 2021: Cyber Attack Dataset Based on RPL Routing for IoT [31] is utilized. The dataset is a comprehensive collection designed to support the development and evaluation of cyberattack detection systems in RPL-based IoT networks. The IoT-RPL 2021 dataset is utilized for experimentation, containing both normal and malicious traffic samples. The dataset includes four attack categories: Blackhole, Flooding, DODAG Version Number, and Decreased Rank attacks. It consists of 24,023 instances generated using the Cooja simulator in a 6LoWPAN-based RPL environment. The dataset reflects realistic attack distributions but exhibits class imbalance with 15,328 attack samples and 1,478 normal samples, reflecting the realistic occurrence of attacks in IoT environments. All data had been generated using the Cooja simulator, which accurately modelled RPL-based IoT networks operating in 6LoWPAN environments.

### Preprocessing stage

Raw data is preprocessed to improve model performance and ensure compatibility with learning algorithms. Initially, categorical features are transformed into numerical representations using one-hot encoding. Subsequently, Pareto scaling normalization [32] is applied to standardize feature distributions while preserving data variance. This step reduces the influence of extreme values and enhances learning stability. This process converts the actual values into the binary series representation, where each exclusive value is indicated as a distinct column or attribute with a value of either 1 or 0. After encoding, data normalization is performed using the Pareto Scaling (PS) technique, which operates similarly to the z-score method but with a key difference: the scaling factor is the square root of the standard deviation (SD). As a result, the newly scaled features will exhibit a variance that matches the standard deviation of the original, unscaled features. Every sample ${y^{\prime }}_{u,m}$ of the data is modified into $y_{u,m}$ and it can be formulated as,


$$\begin{array}{c} {y^{\prime }}_{u,m}=\frac{y_{u,m}-\lambda_{u}}{\sqrt{\sigma_{u}}} \end{array}$$ (1)

Here, $\lambda_{u}$ and $\sigma_{u}$ represents the mean and SD of $u^{th}$ feature respectively.

### Feature selection using the Ch-PKO Technique

The preprocessed data is then fed into the feature selection phase to choose the most relevant feature for minimizing the time consumption and classification error. Metaheuristic optimization strategies are generally used to extract the optimal features and prevent the dimensionality reduction problem. To reduce redundancy and computational overhead, an optimized feature selection mechanism based on the Chaotic Pied Kingfisher Optimizer (Ch-PKO) algorithm is employed. This metaheuristic approach identifies the most relevant features by balancing exploration and exploitation strategies. The algorithm iteratively evaluates candidate feature subsets using a fitness function and selects an optimal subset that maximizes classification performance while minimizing complexity. The PKO algorithm [33] is a novel swarm-based meta-heuristic technique inspired by the unique hunting and cooperative behaviours of pied kingfishers. It operates through three key phases: perching or hovering for target detection, diving for capturing prey, and resting to ensure coexistence. These behaviours are mathematically formulated to enable the PKO to efficiently tackle complex optimization tasks across various search spaces. The approach effectively avoids being trapped in local optima and demonstrates strong global search capabilities.

*Step 1: Initialization Phase*

Similar to other population-based methods, the PKO begins by randomly generating an initial population within the defined search boundaries, as expressed by the following equation (2).


$$\begin{array}{c} P_{x,d}=lb+\left(ub-lb\right)\times rand\text{\textbackslash{}hspace\{0.17em}\;\;\;\}x=1,2,...,M,\text{\textbackslash{}hspace\{0.17em}\;\;\}and\text{\textbackslash{}hspace\{0.17em}\;\;\}d=1,2,....,n \end{array}$$ (2)

Here, $P_{x,d}$ indicates the location of the $n^{th}$ candidate in the dimension d, randsignifies the random values obtained within the interval 0 and 1. Moreover, ub and lbdenotes the upper and lower bounds within the search space, Mand n indicates the total number of candidates and the dimensions, respectively.

In the proposed optimizer, the innovative tent Chaos (TCh) mapping function is utilized that minimize the initialization problems within the search space. The mathematical formulation for the TCh mapping is deliberated below:


$$\begin{array}{c} P_{x,d}=\left\{ \begin{array}{c} @l@P^{l}=2\times a\times P^{i}\text{\textbackslash{}hspace\{0.17em}\;\;\;\;\;\;\;\;\;\;\;\;\;\;\;\;\;\;\;\;\;\;\;\}0\leq P^{i}<\frac{\text{1}}{\text{2}}\\ P^{l}=2\times a\times \left(1-P^{i}\right)\text{\textbackslash{}hspace\{0.17em}\;\;\;\;\;\;\;\}\frac{\text{1}}{\text{2}}\leq P^{i}<1 \end{array}\right. \end{array}$$ (3)

Here, aindicates the control parameter that defines the chaotic mapping and krepresents the number of iterations.

*Step 2: Random Generation*

After initialization, a suitable solution is arbitrarily selected from the pool of optimal parameter sets.

*Step 3: Fitness Function*

The PKO technique utilizes fitness function (FF) for analysing the optimality of the introduced segmentation model, and it is formulated in equation (4).


$$\begin{array}{c} f=max\left(Accuracy\right) \end{array}$$ (4)

*Step 4: Perching/Hovering Strategy (Exploration Phase)*

This stage involves drawing its inspiration from the natural hovering and perching actions observed by PKs, where the birds tactically switch between these actions depending on environmental conditions. In the PKO algorithm, the locations of solution agents are initially determined based on these foraging strategies and are subsequently updated iteratively using the following expression (5).


$$\begin{array}{c} P_{u}\left(i+1\right)=P_{u}\left(i\right)+\beta *K\times \left(P_{v}\left(i\right)-P_{u}\left(i\right)\right)\text{\textbackslash{}hspace\{0.17em}\;\;\;\;\;\}u,v=1,2,....,M\text{\textbackslash{}hspace\{0.17em}\;\;\;\}and\text{\textbackslash{}hspace\{0.17em}\;\;\}u\neq v \end{array}$$ (5)

Here, i interprets the present iteration, $P_{u}\left(i+1\right)$ manipulates the candidate solution in the following iterations, β denotes the parameter which can be formulated as, $\beta =2\times rand_{m}\left(1,n\right)-1$ whereas, $rand_{m}$ signifies the random value under a normal distribution. The parameter K stores the necessary location, and it is flexibly determined based on the present strategy.

At the time of the perching stage, the parameter K can be mathematically indicated as,


$$\begin{array}{c} K=e^{\text{1}}-exp\left(\frac{i-1}{T}\right)*cos\left(z\right) \end{array}$$ (6)

$$\begin{array}{c} z=2*\pi *rand \end{array}$$ (7)

Here, T depicts the maximum iteration. At the time of the hovering phase, the parameter K can be articulated as,


$$\begin{array}{c} K=y*\left(\frac{i^{\frac{\text{1}}{Y}}}{T^{\frac{\text{1}}{Y}}}\right) \end{array}$$ (8)

$$\begin{array}{c} Y=rand*\left(\frac{G\left(v\right)}{G\left(u\right)}\right) \end{array}$$ (9)

Here, G(u) and G(v) interprets the fitness of $u^{th}$ and $v^{th}$ candidate. The parameter Y is a fixed constant and its value is 8.

*Step 5: Diving Strategy (Exploitation Phase)*

The diving behaviour of the PK determines it to be an extremely effective predator, playing a vital role in its survival and success within its ecosystem. This hunting strategy can be mathematically modelled as follows:


$$\begin{array}{c} P_{u}\left(i+1\right)=P_{u}\left(i\right)+Q*o*\beta *\left(y-P_{best}\left(i\right)\right)\text{\textbackslash{}hspace\{0.17em}\;\;\;\;\;\}u=1,2,....M \end{array}$$ (10)

Here, $P_{best}\left(i\right)$ indicates the best location, o and Q presents the hunting performance, which can be interpreted as,


$$\begin{array}{c} Q=rand*\left(\frac{G\left(u\right)}{G}\right) \end{array}$$ (11)

$$\begin{array}{c} o=exp{\left(\frac{-i}{T}\right)}^{\text{2}} \end{array}$$ (12)

Here, G symbolizes the optimal value. The hunting success of the pied kingfisher is shaped by various factors, such as prey availability, individual hunting skills, and the presence of competing or predatory species in the vicinity. During the coexistence stage, this behaviour is represented mathematically as:


$$\begin{array}{c} P_{u}\left(i+1\right)=\left\{\begin{array}{c} @l@P_{u}\left(i\right)+o*\beta *abs\left(P_{u}\left(i\right)-P_{v}\left(i\right)\right)\text{\textbackslash{}hspace\{0.17em}\;\;\;\;\}rand>\left(1-H\right)\\ P_{u}\left(i\right)\text{\textbackslash{}hspace\{0.17em}\;\;\;\;\;\;\;\;\;\;\;\;\;\;\;\;\;\;\;\;\;\;\;\;\;\;\;\;\;\;\;\;\;\;\;\;\;\;\;\;\;\;\;\;\;\;\;\;\;\;\;\;\;\;\;\;\;\;\;\;\;\;\;\;\;\;\;\;\;\;\;\;\;\;\;\;\;\;\}rand\leq \left(1-H\right) \end{array}\right\} \end{array}$$ (13)

$$\begin{array}{c} H=H_{max}-\left(H_{max}-H_{min}\right)*\left(\frac{i}{T}\right) \end{array}$$ (14)

Here, $P_{u}\left(i\right)$ and $P_{v}\left(i\right)$ depicts the position of two arbitrarily determined candidates in the population. The parameter H symbolizes the PK’s predatory efficacy, the values of $H_{max}$ and $H_{min}$ are selected as 0.5 and 0, respectively.

*Step 6: Return the Best Optimal Solution*

*Step 7: Termination criteria*

The proposed PKO method advanced to the next phase, where the updating procedure was iteratively applied using equations (5) to (10) until the stopping criteria were fulfilled. Once the process concluded, the globally optimal features obtained through these repeated iterations were selected and given to the proposed DL model for effective classification. The selected features derived from the Ch-PKO technique included: *frameproof*, *protocol*, *control type*, *type_cont_messg*, *DOAGID*, and *DOAGID.1*, *DOAGID.2*, *DOAGID.3*, *DOAG_info*, *DIO_info*, *object_cont_pt*, *lifetime*, and *prefix_info*. These features collectively captured essential protocol characteristics, control message types, object content points, and lifetime parameters necessary for efficient data representation and accurate modeling. Algorithm 1 depicted the pseudocode for the proposed Ch-PKO technique.

Algorithm 1: Ch-PKO Feature Selection Algorithm

Input:

- Pre-processed dataset D

- Population size N

- Maximum iterations *T*_max_

- Search space bounds [lb, ub]

- Control parameter μ for tent chaos mapping

Output:

- Optimal feature subset

1: BEGIN

2: // Step 1: Initialization Phase with Chaotic Tent Mapping

3: FOR i = 1 to N DO

4: FOR j = 1 to d DO

5:  Generate chaotic value using Tent Chaos mapping:

6:  x_i^j = lb^j + (ub^j – lb^j) × TCh_i

7:  where TCh_i = {μ × TCh_{i-1} if TCh_{i-1} < 0.5

8:    {μ × (1 - TCh_{i-1}) if TCh_{i-1} ≥ 0.5

9:  END FOR

10:    END FOR

11:    // Step 2: Random Generation

12:    Select a random solution from the initialized population

13:    // Step 3: Evaluate Fitness Function

14:    FOR each candidate x_i DO

15:     Calculate fitness: Fitness(x_i) = f(x_i)

16:    END FOR

17:    Identify the best solution x_best with optimal fitness

18:    t = 1 // Initialize iteration counter

19:    WHILE t ≤ T_max DO

20:    FOR each candidate i DO

21:   // Step 4: Perching/Hovering Strategy (Exploration Phase)

22:   Calculate parameter: a = 2 - (2 × t/T_max)

23:   Generate a random value r from the normal distribution

25:   IF random () < 0.5 THEN // Perching phase

26:     x_rand = random candidate from population

27:     P = x_rand + r × a × (x_rand - x_i)

28:   ELSE // Hovering phase

29:    IF Fitness(x_i) <Fitness(x_rand) THEN

30:     P = x_i + r × a × |x_i - x_rand|

31:   ELSE

32:     P = x_rand + r × a × |x_i - x_rand|

33:   END IF

34:    END IF

35:    // Step 5: Diving Strategy (Exploitation Phase)

36:    IF random () < 0.5 THEN

37:        x_i^{t + 1} = x_best - H_1 × |C_1 × x_best – x_i|

38:      where H_1 = 2 × e^{-(Fitness(x_i)/Fitness_optimal) ^8}

39:     C_1 = 2 × random ()

40:   ELSE // Coexistence stage

41:    Select two random candidates x_r1 and x_r2

42:    x_i^{t+1} = x_i + H_2 × (x_r1 - x_r2)

43:    where H_2 = (2 × random () - 0.5) × e^{-(2×t/T_max) ^2}

44:   END IF

45:   // Boundary constraint handling

46:   x_i^{t+1} = clip(x_i^{t+1}, lb, ub)

47:   // Evaluate new fitness

48:   Calculate Fitness(x_i^{t + 1})

49:   // Update best solution

50:   IF Fitness(x_i^{t + 1}) <Fitness(x_best) THEN

51:    x_best = x_i^{t+1}

52:   END IF

53:    END FOR

54:    t = t + 1 // Increment iteration

55:    END WHILE

56:    // Step 6: Return Best Optimal Solution

57:    Extract selected features from x_best:

58: Selected_Features = {frameproof, protocol, control_type,

59:     type_cont_messg, DOAGID, DOAGID.1, DOAGID.2,

60:     DOAGID.3, DOAG_info, DIO_info, object_cont_pt,

61:     lifetime, prefix_info}

62:    // Step 7: Termination

63:    RETURN Selected_Features

64:    END

### RPL attack detection using the ZCAtt-BiLSTM technique

The selected features are provided as input to the Zero-parameter Channel Attention Bidirectional Long Short-Term Memory (ZCAtt-BiLSTM) model. The BiLSTM component [34] captures temporal dependencies in network traffic by processing sequences in both forward and backward directions. To enhance feature discrimination and prevent overfitting, a zero-parameter channel attention module is integrated. This module highlights informative features without introducing additional trainable parameters, improving classification efficiency and accuracy. The detailed analysis of the proposed framework is discussed below:

The LSTM is a diverse subtype of RNNs utilized for progressive data processing. The LSTM consists of two essential components: a gating unit (GU) and a memory unit (MU). The MU helps to store and transfer data, and the GU maintains the data flow regulation. The input gates (IG) deliberate the concern over transmitting new input into the MU. Simultaneously, the tanh function is computed to obtain vectors, thereby upgrading the parameters within the MU. By utilizing the sigmoid function and previous memory states, this gate produces a value between the range 0 and 1. The outcome gate is assigned to discriminate the memory cell segments to send the corresponding hidden state. The mathematical expressions for the LSTM training process are deliberated in equations (5–9),


$$\begin{array}{c} x_{k}=\sigma \left(W_{ix}i_{k}+W_{ih}h_{k-1}+W_{id}d_{k-1}+Bias_{x}\right), \end{array}$$ (15)

$$\begin{array}{c} f_{k}=\sigma \left(W_{if}i_{k}+W_{hf}h_{k-1}+W_{fd}d_{k-1}+Bias_{f}\right), \end{array}$$ (16)

$$\begin{array}{c} d_{k}=f_{k}d_{k-1}+x_{k}tanh\left(W_{id}i_{k}+W_{hd}h_{k-1}+Bias_{d}\right), \end{array}$$ (17)

$$\begin{array}{c} y_{k}=\sigma \left(W_{yx}i_{k}+W_{yh}h_{k-1}+W_{yd}d_{k}+Bias_{y}\right), \end{array}$$ (18)

$$\begin{array}{c} h_{k}=y_{k}tanh\left(d_{k}\right) \end{array}$$ (19)

Here, σ indicates the sigmoid function, x, f, d, and y represents the IG, forget gate, activated cell vector, and the output gate, respectively. Moreover, h and W represents the hidden trajectory, and the weights between two units, respectively. However, there arises some complexity in accessing previous state information using the LSTM model. To tackle this issue, a BiLSTM model that utilizes both forward and backward LSTMs to process the incoming features in opposite directions.

**Fig 3:**
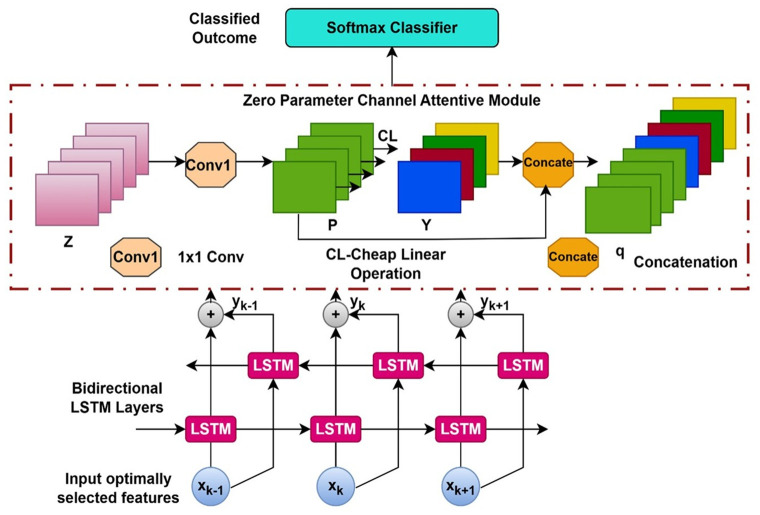
Architecture of proposed ZCAtt-BiLSTM technique.

This bidirectional technique optimally controls the data based on the current time step. This wide-ranging perception assists in determining active patterns and bears the long-term dependencies over attack samples. Initializing the training process, the sequential feature subsets are encompassed in the BiLSTM model. The sequence lengths are coordinated at the input layer to ensure uniformity. This process aids the sequential inputs with similar time steps, resulting in $y_{1,}y_{\text{2}},.....y_{n}$ have similar time steps after being utilized as the input. The flow of samples regulates the memory updates via the LSTM layer, enabling BiLSTM to integrate both backward and forward directions. Fig 3 depicts the architecture of the proposed ZCAtt-BiLSTM technique.

*• Zero Channel Attentive (ZCAtt) Module*

The aim of the ZCAtt module in cyberattack detection is to determine the region where the abnormal data is from the feature map. Linear transform increases the necessity of final channels. Particularly, it calculates the linear separability between the final and other channels according to the energy function as,


$$\begin{array}{c} E=\frac{4\left(\sigma^{\text{2}}+\beta \right)}{{\left(g_{c}-\rho \right)}^{\text{2}}+2\sigma^{\text{2}}+2\beta } \end{array}$$ (20)

Here, ρ and $\sigma^{\text{2}}$ indicates the mean and variance of the feature $g_{c}$, β manipulates the constant to eliminate σ to move into the value 0, and it is set to the value 0.0001. The above equation indicates that the energy function is defined using the linear transformation of mean and variance for the spatial features of every channel. The minimal value of E, the channel obtained will be more discriminated from the nearby channel; hence, the maximum weight is to be determined. The reciprocal of equation (3) deliberates the necessity of the dataset.

The ZCAtt module is introduced to enhance the detection performance without increasing the parameters. Assume an input P, the global average pooling process is initially trained to minimize the size of P to 1×1 which generates the set of global attention maps N. Using the energy function, the updated channel attention maps M are successively obtained by utilizing the sequential element-wise operation on N. It can be formulated as,


$$\begin{array}{c} N=\sum_{a=1}^{A}\frac{{\left(M_{a}-\rho \right)}^{\text{2}}+2\left(\sigma^{\text{2}}+\beta \right)}{4\left(\sigma^{\text{2}}+\beta \right)} \end{array}$$ (21)

Here, $M_{a}$ indicates the $a^{th}$ CAtt map. In the next phase, the attention map M is remodified using the sigmoid function. In the final stage, Pis multiplied by M to generate the outcome q. The function of the ZCAtt module can be expressed as,


$$\begin{array}{c} q=P\times \left\langle sig\left(g_{e}\left(GAP\left(P\right)\right)\right)\right\rangle \end{array}$$ (22)

Here, GAP and $g_{e}$ indicates the global average pooling and linear transformation process, respectively. The ZCAtt module has no training parameters, and the enhanced channel feature maps can be generated using mathematical operations.

### Performance metrics

#### Accuracy (%).

Accuracy represented the overall correctness of a model, in terms of how often it correctly predicted both positive and negative outcomes. It was a general indicator of model performance, but was potentially misleading when the data was imbalanced.


$$\begin{array}{c} Accuracy=\frac{TP+TN}{TP+TN+FP+FN}\times \text{1}\text{0}\text{0} \end{array}$$

#### Precision (%).

Precision measured how reliable the model’s positive predictions were. A high precision meant that when the model predicted a positive, it was usually correct.


$$\begin{array}{c} Precision=\frac{TP}{TP+FP}\times \text{1}\text{0}\text{0} \end{array}$$

#### Recall (%).

Recall (or Sensitivity) measures the model’s ability to correctly identify actual positives. It showed how many of the true positive cases were captured by the model.


$$\begin{array}{c} Recall=\frac{TP}{TP+FN}\times \text{1}\text{0}\text{0} \end{array}$$

#### Specificity (%).

Specificity quantified how well the model identified true negatives. It was especially important in applications where false positives had to be minimized.


$$\begin{array}{c} Specificity=\frac{TN}{TN+FP}\times \text{1}\text{0}\text{0} \end{array}$$

#### F1-Score (%).

The F1-score was the harmonic mean of precision and recall. It provided a balanced measure that considered both false positives and false negatives.


$$\begin{array}{c} F\text{1}-Score=\text{2}\times \frac{Precision\times Recall}{Precision+Recall}\times \text{1}\text{0}\text{0} \end{array}$$

#### F2-Score (%).

The F2-score was similar to the F1-score but gave more weight to recall. It was useful in situations where missing positives (false negatives) are more critical.


$$\begin{array}{c} F\text{2}-Score=\left(\text{1}+{\text{2}}^{\text{2}}\right)\times \frac{Precision\times Recall}{\left({\text{2}}^{\text{2}}\times Precision\right)+Recall}\times \text{1}\text{0}\text{0} \end{array}$$

#### G-Mean (%).

The Geometric Mean (G-Mean) balanced sensitivity and specificity. It ensured that both positive and negative classes were predicted with equal importance.


$$\begin{array}{c} G-Mean=\sqrt{Recall\times Specificity}\times \text{1}\text{0}\text{0} \end{array}$$

#### Balanced Accuracy (%).

Balanced accuracy was the average of recall (sensitivity) and specificity. It was more reliable than raw accuracy for imbalanced datasets.


$$\begin{array}{c} BalancedAccuracy=\frac{Recall+Specificity}{\text{2}}\times \text{1}\text{0}\text{0} \end{array}$$

#### Matthews Correlation Coefficient (MCC).

MCC measured the correlation between predicted and actual classifications. It considered all confusion matrix values and provided a balanced score even for imbalanced datasets.


$$\begin{array}{c} MCC=\frac{\left(TP\times TN)-(FP\times FN\right)}{\sqrt{\left(TP+FP)(TP+FN)(TN+FP)(TN+FN\right)}} \end{array}$$

#### Kappa coefficient (Cohen’s Kappa).

Cohen’s Kappa evaluated inter-rater agreement between the predicted and actual classifications, adjusting for agreement that occurred by chance.


$$\begin{array}{c} \kappa =\frac{P_{o}-P_{e}}{\text{1}-P_{e}} \end{array}$$

where *P*_*o*_  = observed accuracy, and $P_{e}$ = expected accuracy by chance.

#### False Discovery rate (FDR %).

FDR quantified the proportion of predicted positives that were actually false. It measured how often the model incorrectly identified negatives as positives.


$$\begin{array}{c} FDR=\frac{FP}{TP+FP}\times \text{1}\text{0}\text{0} \end{array}$$

#### False Positive Rate (FPR %).

FPR indicated how frequently actual negatives were incorrectly classified as positives. It complemented specificity (FPR = 1 – Specificity).


$$\begin{array}{c} FPR=\frac{FP}{FP+TN}\times \text{1}\text{0}\text{0} \end{array}$$

#### False Negative Rate (FNR %).

FNR measured how many actual positives were missed by the model, cases where the model failed to detect a true positive.


$$\begin{array}{c} FNR=\frac{FN}{TP+FN}\times \text{1}\text{0}\text{0} \end{array}$$

#### Negative Predictive Value (NPV %).

NPV measured the probability that subjects predicted as negative were truly negative. It helped evaluate the reliability of negative predictions.


$$\begin{array}{c} NVP=\frac{TN}{TN+FN}\times \text{1}\text{0}\text{0} \end{array}$$

#### Detection Rate (%).

The detection rate was equivalent to the recall or Sensitivity. It represented the percentage of actual positive cases correctly identified by the model.


$$\begin{array}{c} DetectionRate=\frac{TP}{TP+FN}\times \text{1}\text{0}\text{0} \end{array}$$

#### AUC-ROC (%).

The Area Under the ROC Curve (AUC-ROC) measured the model’s ability to distinguish between classes across all possible thresholds. Higher AUC indicated better discrimination.


$$\begin{array}{c} AUC-ROC=\int_{\text{0}}^{\text{1}}TPR(FPR)d(FPR)\times \text{1}\text{0}\text{0} \end{array}$$

#### AUC-PR (%).

The Area Under the Precision-Recall Curve (AUC-PR) summarized the trade-off between precision and recall for different thresholds. It was particularly useful for imbalanced datasets.


$$\begin{array}{c} AUC-PR=\int_{\text{0}}^{\text{1}}Precision(Recall)d(Recall)\times \text{1}\text{0}\text{0} \end{array}$$

## Results and discussion

This section presents the experimental results of the proposed work to detect intrusion. The reproducibility ensured that all experiments were conducted in a controlled software environment. The DL backend, as TensorFlow and Keras with Python programming language, implements the proposed model. The proposed method is processed under Intel(R) Core (TM) i5-4300M CPU with 4GB installed RAM using a 64-bit operating system. For the training process, 80% of the training data, 10% of the testing data, and 10% of the validation data are considered, which are in the ratio 8:1:1. Table 1 shows the parameter settings for the proposed study.

**Table 1:** Proposed parameter configurations.

Parameters		Ranges
**Ch-PKO**	Inertia weight	0.9
	Chaotic map	Logistic map
	Max iterations	100
	Population size	30
	Fitness function	Weighted F1-score
	Acceleration coefficients	2.0
**ZCAtt-BiLSTM model**	BiLSTM layers	2
	BiLSTM hidden units	128
	Epochs	100
	Batch size	64
	Dropout rate	0.3
	Learning rate	0.001

**Fig 4:**
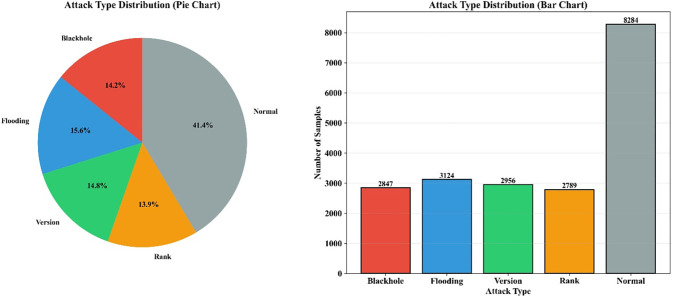
The dataset’s class distribution.

Fig 4 provided a comprehensive overview of the dataset’s class distribution, utilizing both a pie chart for relative percentages and a bar chart for absolute sample counts. The data was categorized into five classes: “Normal” and four distinct attack types (“Blackhole,” “Flooding,” “Version,” and “Rank”). A significant class imbalance was immediately apparent from both visualizations. The “Normal” class constituted the clear majority, accounting for 41.4% of the dataset, which the bar chart quantified as 8284 samples. The four attack classes, while being minority classes relative to “Normal,” were distributed in a fairly balanced manner among themselves. “Flooding” was the most frequent attack (15.6% or 3124 samples), followed closely by “Version” (14.8% or 2956 samples), “Blackhole” (14.2% or 2847 samples), and “Rank” (13.9% or 2789 samples). This distribution underscored the challenge of the classification task, as any effective model had to perform well on both the dominant “Normal” class and the less frequent, yet critically important, attack classes.

**Fig 5:**
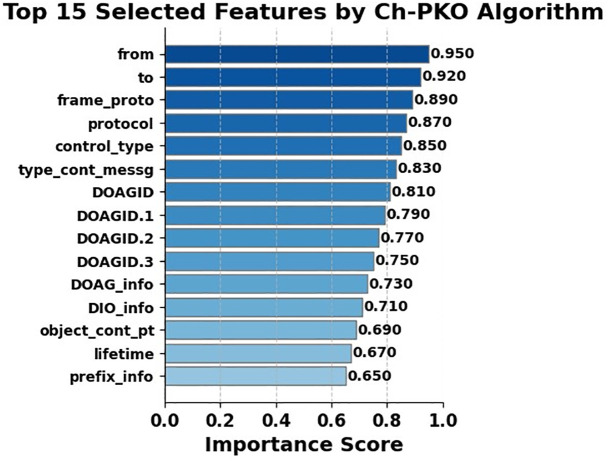
Feature selection process.

Fig 5 illustrates the results of the feature selection process conducted using the “Ch-PKO Algorithm,” displaying the top 15 most discriminative features ranked by their “Importance Score.” The horizontal bar chart clearly ranked features from most to least influential, with scores ranging from 0.0 to 1.0. At the very top, the ‘from’ and ‘to’ features were identified as the most critical, with high importance scores of 0.950 and 0.920, respectively. These were closely followed by other high-level protocol identifiers, such as ‘frame_proto’ (0.890), ‘protocol’ (0.870), and ‘control_type’ (0.850). The list also included a cluster of related “DOA” features (e.g., ‘DOAGID,’ ‘DOAGID.1,’ ‘DOAG_info’) as well as temporal features like ‘lifetime’ (0.670), all of which contributed significantly to the model’s predictive power. The chart showed a smooth, gradual decline in importance down to the 15th feature, ‘prefix_info’ (0.650), providing a clear, prioritized subset of data for the subsequent modelling phase.

**Fig 6:**
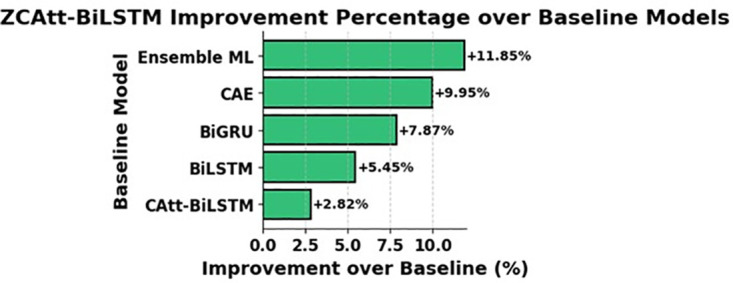
Performance gain.

Fig 6 presents the performance gain of the proposed “ZCAtt-BiLSTM” model relative to five other baseline models. The y-axis listed the baseline models against which the comparison was made, while the x-axis measured the “Improvement over Baseline (%)” in terms of overall accuracy. A clear trend was visible: the magnitude of improvement was inversely proportional to the performance of the baseline itself. The most significant gain was observed over the “Ensemble ML” model, with a substantial +11.85% improvement. This was followed by a + 9.95% improvement over “CAE” and +7.87% over “BiGRU.” Even when compared to the next-best-performing model, “CAtt-BiLSTM,” the proposed method still demonstrated a noteworthy advantage of +2.82%, highlighting its consistent and measurable superiority across the entire model spectrum.

**Fig 7:**
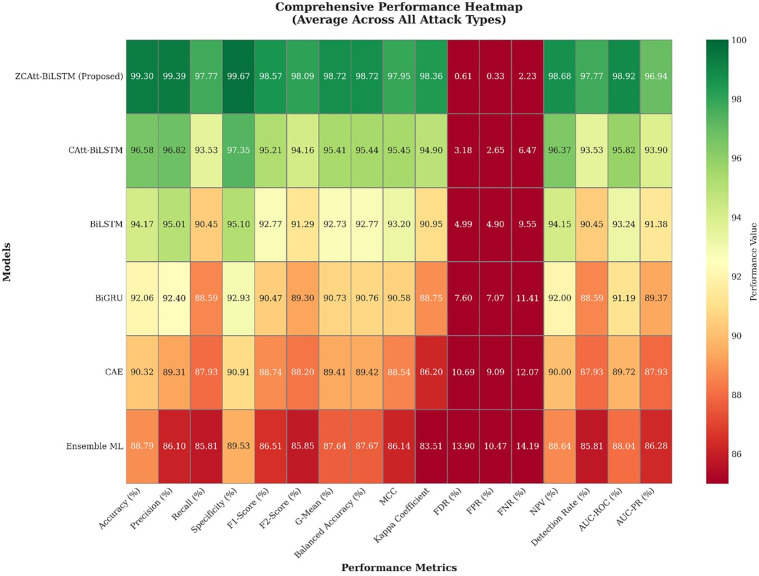
Performance heatmap.

Fig 7 presents a comprehensive performance heatmap, meticulously detailing a comparative analysis of six distinct computational models based on their average performance across various attack types. The models, listed vertically, included “Ensemble ML,” “CAE,” “BiGRU,” “BiLSTM,” “CAtt-BiLSTM,” and a “ZCAtt-BiLSTM (Proposed)” model. These were evaluated against seventeen different performance metrics, displayed horizontally, which covered a wide spectrum of evaluative criteria such as Accuracy, Precision, Recall, F1-Score, MCC, Kappa Coefficient, AUC-ROC, and various error rates like FPR and FNR. The heatmap employed a colour gradient where deep green represented a high-performance value (near 100%) and deep red signified a lower performance value (near 85%). It was immediately apparent that the “ZCAtt-BiLSTM (Proposed)” model demonstrated overwhelmingly superior performance, as its entire row was dominated by dark green cells, with most metrics achieving scores above 98%. Conversely, performance clearly degraded down the list, with the “Ensemble ML” model exhibiting the weakest results, characterized by numerous red and orange cells. A key observation was in the error-rate columns (FDR, FPR, FNR), where the proposed model achieved the lowest (best) values, such as 0.33% FPR, which, despite the fixed colour scale, were vastly superior to the double-digit error rates seen in the baseline models.

**Fig 8:**
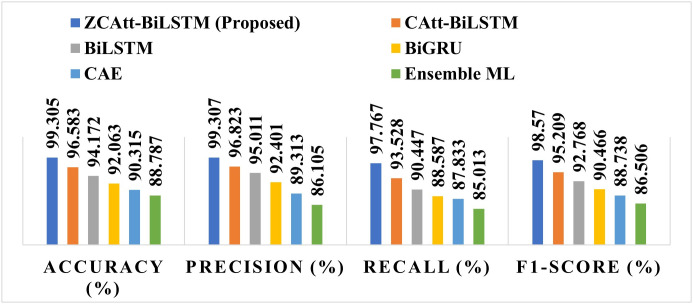
Model comparison visualization.

Fig 8 and Table 2 showed the models being evaluated were the “ZCAtt-BiLSTM (Proposed),” “CAtt-BiLSTM,” “BiLSTM,” “BiGRU,” “CAE,” and “Ensemble ML.” The evaluation was based on four primary classification performance metrics: Accuracy, Precision, Recall, and F1-Score, all presented as percentages. A striking and consistent trend was immediately discernible from the chart: the “ZCAtt-BiLSTM (Proposed)” model unequivocally outperformed all five counterparts across every metric. It achieved the highest scores in Accuracy (99.305%), Precision (99.307%), Recall (97.767%), and F1-Score (98.57%). Following the proposed model, a clear performance hierarchy was established, with “CAtt-BiLSTM” serving as the second-best, and performance generally degrading through “BiLSTM,” “BiGRU,” and “CAE.” The “Ensemble ML” model consistently registered the lowest performance, for example, achieving only 88.787% accuracy and 86.506% in F1-Score, thereby highlighting the significant performance gains of the proposed architecture.

**Table 2:** Model comparison over the performance metrics like Accuracy, Precision, Recall, and F1-Score.

Metric	ZCAtt-BiLSTM (Proposed)	CAtt-BiLSTM	BiLSTM	BiGRU	CAE	Ensemble ML
**Accuracy (%)**	99.305	96.583	94.172	92.063	90.315	88.787
**Precision (%)**	99.307	96.823	95.011	92.401	89.313	86.105
**Recall (%)**	97.767	93.528	90.447	88.587	87.833	85.013
**F1-Score (%)**	98.57	95.209	92.768	90.466	88.738	86.506

**Fig 9:**
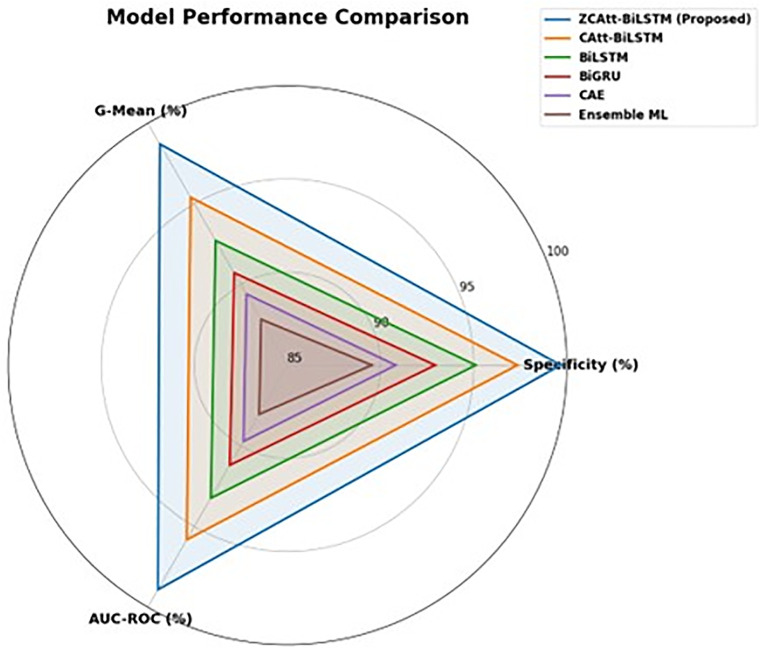
Comparison of the model’s visualization in a radar chart.

Fig 9 and Table 3 present a model performance comparison using a radar chart, supplemented by a precise data table, to evaluate six distinct models: “ZCAtt-BiLSTM (Proposed),” “CAtt-BiLSTM,” “BiLSTM,” “BiGRU,” “CAE,” and “Ensemble ML.” The comparison was plotted along three critical performance axes: Specificity (%), G-Mean (%), and AUC-ROC (%). The chart’s concentric layout, with values scaling from 85% to 100%, provided an immediate and clear visualization of relative performance. The “ZCAtt-BiLSTM (Proposed)” model, represented by the outermost blue triangle, decisively outperformed all other models by encompassing the largest area, indicating its superior scores on all three metrics. A distinct, nested performance hierarchy was evident, with the ‘CAtt-BiLSTM’ model (orange) performing second best, followed sequentially by ‘BiLSTM’ (green), ‘BiGRU’ (red), and ‘CAE’ (purple). The “Ensemble ML” model (brown) formed the innermost triangle, signifying the lowest performance in this comparative set. The accompanying table corroborated this visual hierarchy with exact figures, quantifying the proposed model’s lead with top scores of 99.674% in Specificity, 98.715% in G-Mean, and 98.915% in AUC-ROC, underscoring its comprehensive superiority.

**Table 3:** Model comparison over the performance metrics of Specificity and G-mean, AUC-ROC.

Metric	ZCAtt-BiLSTM (Proposed)	CAtt-BiLSTM	BiLSTM	BiGRU	CAE	Ensemble ML
**Specificity (%)**	99.674	97.346	95.103	92.932	90.811	89.531
**G-Mean (%)**	98.715	95.407	92.726	90.727	89.409	87.843
**AUC-ROC (%)**	98.915	95.819	93.24	91.194	89.72	88.044

**Fig 10:**
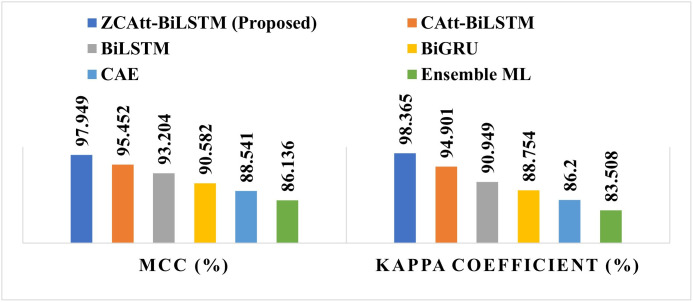
Model comparison visualization.

Table 4 and Fig 10 present a comparative performance analysis of six models using two robust statistical metrics: the Matthews Correlation Coefficient (MCC) and the Kappa Coefficient, both expressed as percentages. The models under investigation, “ZCAtt-BiLSTM (Proposed),” “CAtt-BiLSTM,” “BiLSTM,” “BiGRU,” “CAE,” and “Ensemble ML,” were evaluated in two distinct grouped bar charts, with the precise values detailed in the accompanying table. A clear and consistent performance hierarchy emerged from the data. The “ZCAtt-BiLSTM (Proposed)” model demonstrated marked superiority, achieving the highest scores in both MCC (97.949%) and Kappa Coefficient (98.365%). This performance was followed in descending order by the “CAtt-BiLSTM” model, with the “Ensemble ML” model registering the lowest performance in both categories (86.136% MCC and 83.508% Kappa), thereby visually and numerically substantiating the significant performance advantage of the proposed model.

**Table 4:** Model comparison over performance metrics MCC, Kappa coefficient.

Metric	ZCAtt-BiLSTM (Proposed)	CAtt-BiLSTM	BiLSTM	BiGRU	CAE	Ensemble ML
**MCC (%)**	97.949	95.452	93.204	90.582	88.541	86.136
**Kappa Coefficient (%)**	98.365	94.901	90.949	88.754	86.2	83.508

**Fig 11:**
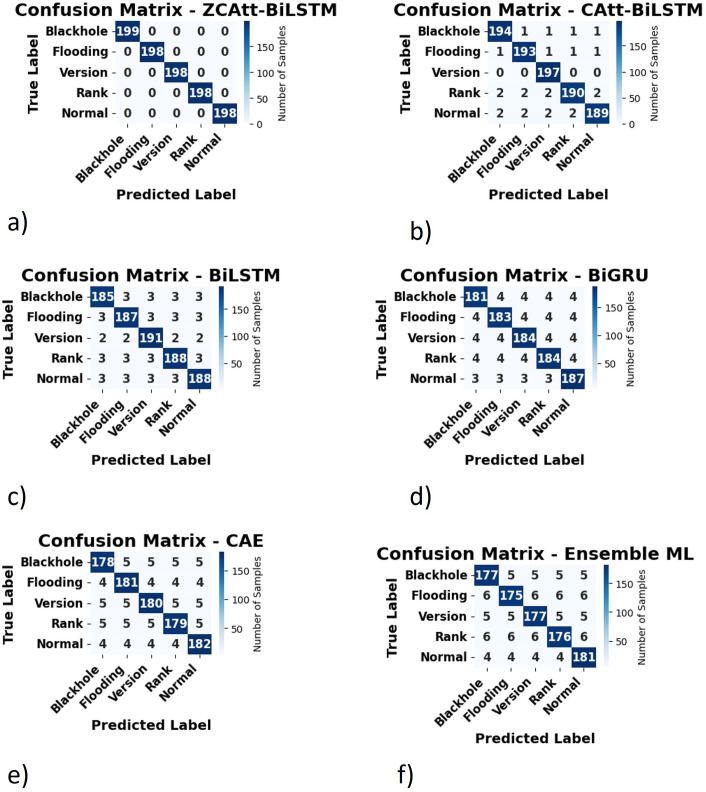
Confusion matrix comparison of different models.

The six distinct confusion matrices in Fig 11 (a-f) provided a granular visualization of the classification performance for each of the evaluated models: ZCAtt-BiLSTM (Proposed), CAtt-BiLSTM, BiLSTM, BiGRU, CAE, and Ensemble ML. All matrices mapped the model’s “Predicted Label” against the “True Label” across five classes: Blackhole, Flooding, Version, Rank, and Normal. The diagonal cells, heavily shaded in dark blue, represented the counts of correct classifications (True Positives), while all off-diagonal cells represented misclassifications. A comparative inspection revealed a stark difference in performance. The ZCAtt-BiLSTM matrix demonstrated a near-perfect classification, with all samples residing on the main diagonal and zero misclassifications in any off-diagonal cell. This indicated exceptional precision and recall for every single class. In contrast, all other models exhibited varying degrees of confusion. The CAtt-BiLSTM matrix showed very strong performance, but with minor misclassifications, such as 2 “Rank” samples being misidentified as “Blackhole” or “Flooding.” The performance degraded further in the BiLSTM and BiGRU matrices, which displayed a more distributed pattern of errors across multiple classes. Finally, the CAE and Ensemble ML matrices showed the weakest performance, characterized by the highest counts in off-diagonal cells; for instance, the Ensemble ML model frequently misclassified 5 or 6 samples from one class as another, indicating significant confusion between the different categories. Collectively, these matrices visually substantiated the profound superiority of the proposed ZCAtt-BiLSTM model, which achieved flawless class discrimination where all other models faltered.

**Fig 12:**
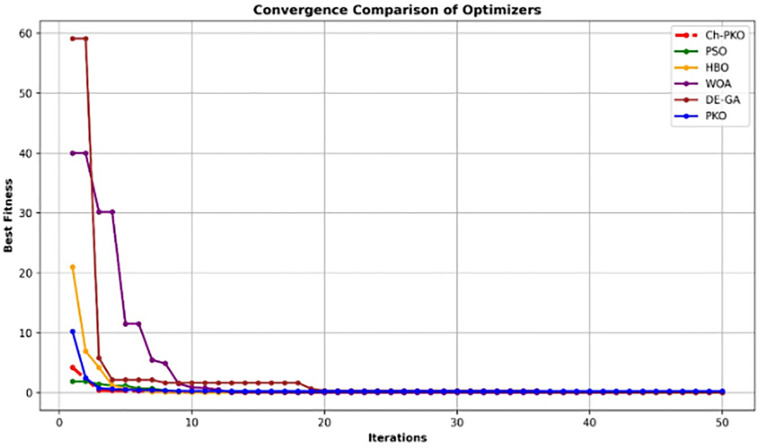
Convergence comparison.

Fig 12 presents a comparative convergence analysis of six distinct optimization algorithms, plotting the “Best Fitness” value against the number of “Iterations” up to 50. The graph illustrated a minimization problem, where the objective was to find the lowest possible fitness value. The six optimizers shown, Ch-PKO, PSO, HBO, WOA, DE GA, and PKO, exhibited markedly different convergence behaviours. The DE GA and WOA algorithms, for instance, began with very high initial fitness values (around 60 and 40, respectively) and demonstrated a slower, stepped descent, requiring 15–20 iterations to reach the optimal solution. In sharp contrast, the Ch-PKO (red), PSO (green), and HBO (orange) algorithms displayed superior performance, starting from much lower initial fitness values and converging exceptionally fast, stabilizing at a “Best Fitness” value near zero in fewer than 10 iterations. The PKO algorithm (blue) also performed well, though it converged slightly slower than the Ch-PKO variant, solidifying the Ch-PKO as one of the most efficient optimizers in this comparison.

**Fig 13:**
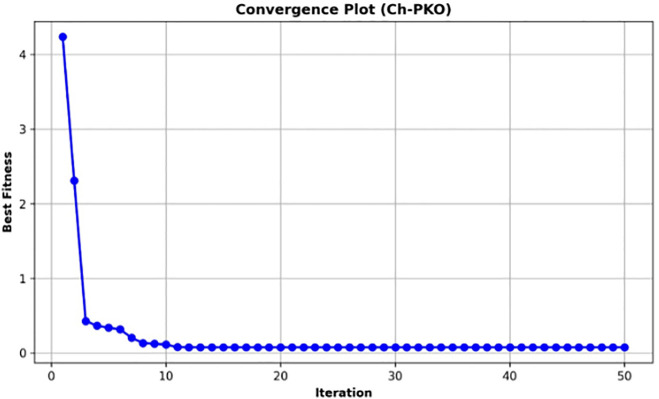
Convergence plot of Ch-PKO.

Fig 13 provided a detailed, magnified view of the convergence trajectory for the " Ch-PKO” optimizer, which was identified as a top performer in the previous comparative chart. By rescaling the “Best Fitness” axis to a maximum of just over 4.0, this plot offered a granular look at the algorithm’s initial optimization phase. The process began with a “Best Fitness” of approximately 4.2 at iteration 1. It then demonstrated an extremely rapid and effective search, with the fitness value plummeting to below 0.5 within the first 5 iterations alone. Following this initial, aggressive descent, the algorithm continued to make finer, incremental improvements until it fully converged to its final, optimal fitness value of near zero by approximately iteration 15, after which it remained stable for the rest of the 50-iteration run.

**Fig 14:**
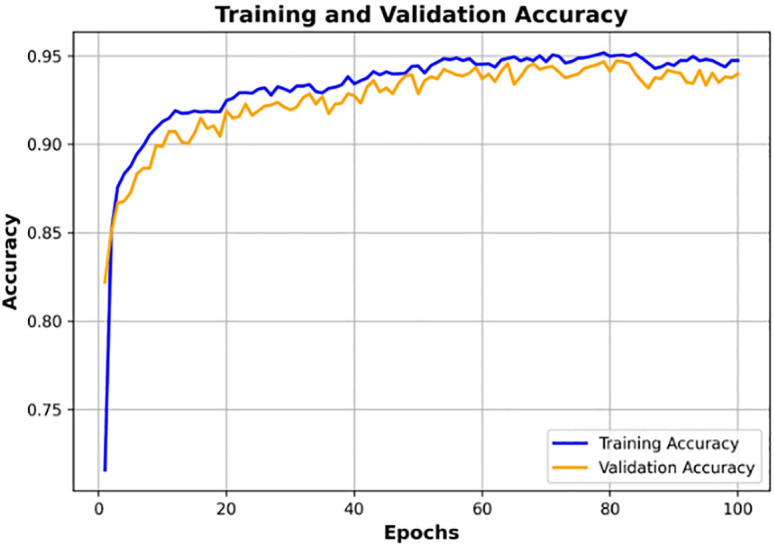
Training and Validation Accuracy.

The graph in Fig 14, which showed “Training and Validation Accuracy,” demonstrated a classic, healthy learning curve. Both the training accuracy (blue line) and validation accuracy (orange line) exhibited a very rapid increase within the first 20 epochs, rising from a low initial state to approximately 90%. Following this steep ascent, the model’s improvement slowed, and both accuracy curves entered a phase of finer-grained optimization, gradually climbing to and fluctuating around the 94–95% mark. Crucially, the validation accuracy closely tracked the training accuracy throughout the entire process, indicating that the model was generalizing well to unseen data and was not suffering from significant overfitting.

**Fig 15:**
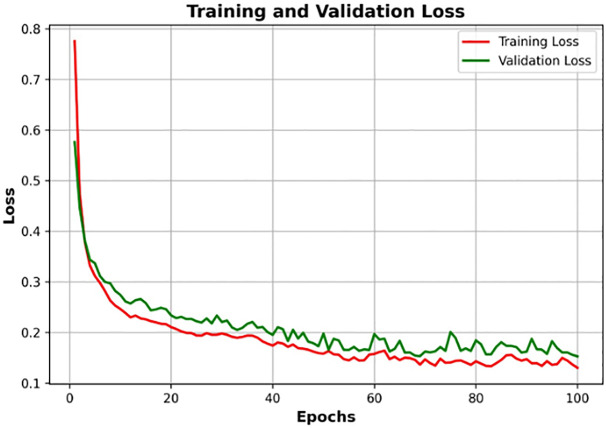
Training and Validation Loss.

The graph in Fig 15, which showed “Training and Validation Loss,” provided a reciprocal perspective on the same training process. It showed a precipitous drop in both training loss (red line) and validation loss (green line) during the initial epochs, corresponding perfectly to the rapid accuracy gains. As the model converged, the rate of loss reduction flattened, with the training loss consistently decreasing to a final value near 0.1. The validation loss mirrored this downward trend, albeit with more high-frequency oscillations and settling at a slightly higher value (around 0.15). This persistent decrease in validation loss, in concert with the validation accuracy, reinforced the conclusion that the model had learned robust patterns rather than simply memorizing the training data.

**Fig 16:**
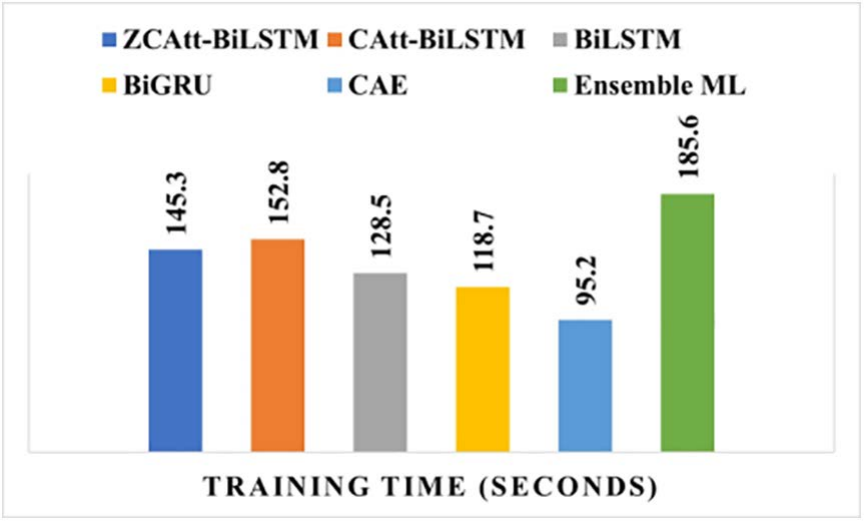
Training time comparison.

Fig 16 shows a comparative analysis of the computational overhead for six different models, specifically measuring their respective Training Time in seconds. The models under evaluation were the “ZCAtt-BiLSTM,” “CAtt BiLSTM,” “BiLSTM,” “BiGRU,” “CAE,” and an “Ensemble ML.” Unlike metrics where higher values were superior, this chart evaluated computational efficiency, where lower times were preferable. It was immediately evident that the “Ensemble ML” model was the most computationally demanding, registering the longest training time at 185.6 seconds. Conversely, the “CAE” model was the most efficient, requiring only 95.2 seconds. The other models fell in between, with the recurrent architectures “BiGRU” (118.7 s) and “BiLSTM” (128.5 s) showing moderate costs. Interestingly, the “ZCAtt-BiLSTM” (145.3 s) and “CAtt BiLSTM” (152.8 s) models, which demonstrated high accuracy in other analyses, also carried a significant computational burden, ranking as the third and second most time-intensive, respectively. This suggested a classic trade-off between model complexity, performance, and the computational resources required for training.

**Fig 17:**
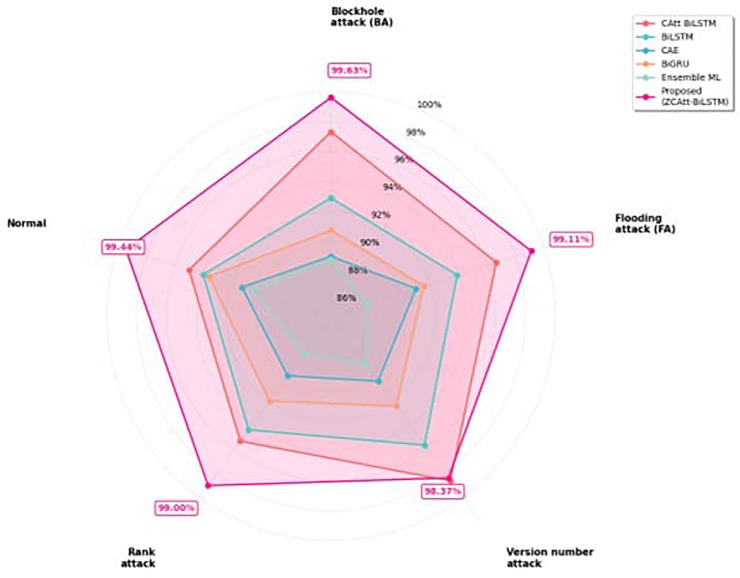
Simulation visualization analysis of the accuracy metric across different methods.

This radar chart (Fig 17) and the corresponding data in Table 5 present a granular class-wise accuracy comparison across six evaluated models: Proposed (ZCAtt-BiLSTM), CAtt-BiLSTM, BiLSTM, CAE, BiGRU, and Ensemble ML. The radar chart provided an immediate visual assessment, with each spoke representing one of the five classes (Blackhole, Flooding, Version Number, Rank, and Normal). The Proposed (ZCAtt-BiLSTM) model, represented by the magenta area, decisively outperformed all baselines by occupying the largest area and achieving the highest accuracy on every single class. Specifically, the proposed model registered near-perfect accuracy, with the highest score achieved on Blackhole attack (99.631%), followed closely by Normal (99.443%) and Flooding attack (99.114%). In contrast, the performance hierarchy among the baselines followed the same pattern observed in previous analyses, with CAtt-BiLSTM generally performing the best (e.g., 97.332% for BA) and the Ensemble ML model consistently registering the weakest class-wise scores (e.g., 87.543% for FA). The significant separation between the magenta boundary and all inner polygons, visually and numerically substantiated the superior classification capability of the proposed ZCAtt-BiLSTM model for all specific attack types and the normal class

**Table 5:** Simulation analysis of the accuracy metric across different methods.

Classes	CAtt BiLSTM	BiLSTM	CAE	BiGRU	Ensemble ML	Proposed (ZCAtt-BiLSTM)
Blackhole Attack (BA)	97.332%	92.910%	89.003%	90.741%	88.784%	99.631%
Flooding Attack (FA)	96.651%	93.892%	90.993%	91.551%	87.543%	99.114%
Version Number Attack	98.601%	95.663%	90.374%	92.431%	88.993%	98.373%
Rank Attack	95.331%	94.392%	89.933%	92.001%	88.113%	99.002%
Normal	94.999%	94.001%	91.273%	93.591%	90.503%	99.443%

**Fig 18:**
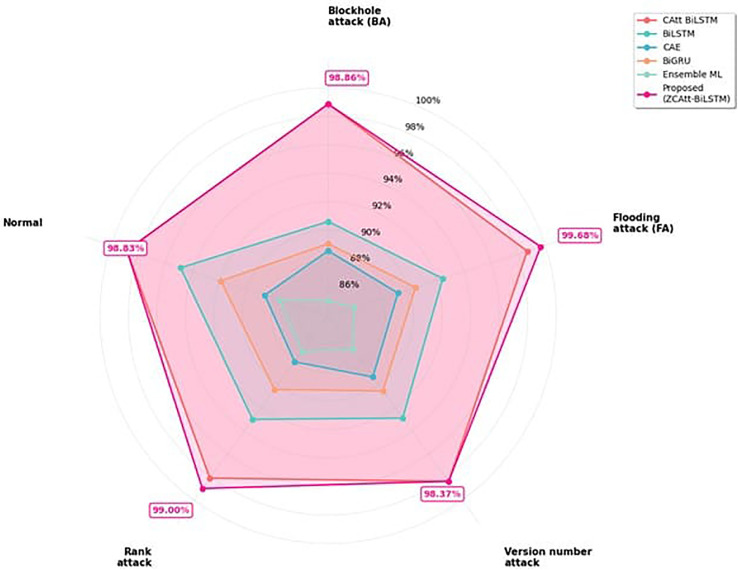
Simulation visualization analysis of the F-score metric across different methods.

Table 6 and Fig 18 presented a class-wise comparison of the F-score metric, a crucial measure of classification accuracy that balanced both precision and recall. The analysis evaluated the Proposed (ZCAtt-BiLSTM) model against five baselines (CAtt-BiLSTM, BiLSTM, CAE, BiGRU, and Ensemble ML) across all five classes (Blackhole, Flooding, Version Number, Rank, and Normal). The Proposed (ZCAtt-BiLSTM) model decisively outperformed all competitors, achieving the highest F-score in every single category, with a peak performance of 99.677% on the Flooding attack (FA) class. Furthermore, the proposed model registered the lowest performance variance across the attack classes, demonstrating an F-score consistency between 98.373% (for Version number attack) and 99.677% (for Flooding attack). In contrast, the baseline models showed significantly lower and more inconsistent results; for example, the Ensemble ML model consistently registered the weakest performance, with its F-scores ranging from a low of 85.051% (for BA) to 87.562% (for Normal), thereby visually and numerically substantiating the superior reliability and balance of the proposed architecture.

**Table 6:** Simulation analysis of the F-score metric across different methods.

Classes	CAtt BiLSTM	BiLSTM	CAE	BiGRU	Ensemble ML	Proposed (ZCAtt-BiLSTM)
Blackhole Attack (BA)	98.864%	90.613%	88.553%	89.054%	85.051%	98.864%
Flooding Attack (FA)	98.677%	92.451%	89.144%	90.444%	85.903%	99.677%
Version Number Attack	98.373%	92.891%	89.324%	90.531%	86.902%	98.373%
Rank Attack	98.102%	93.002%	87.999%	92.001%	87.110%	99.002%
Normal	98.832%	94.882%	88.672%	93.591%	87.562%	98.832%

Table 7 presented a class-wise comparison of the False Discovery Rate (FDR) metric, which quantified the percentage of false positive predictions and, unlike accuracy metrics, indicated model failure (lower values were superior). The analysis evaluated the Proposed (ZCAtt-BiLSTM) model against five baselines across all five attack and normal classes. The Proposed (ZCAtt-BiLSTM) model decisively demonstrated the lowest FDR across every category, signifying its unparalleled ability to minimize false alarms. Specifically, the proposed model achieved sub-1% FDR across four of the five classes, with its best performance registered on the Blackhole attack (0.0987%). In stark contrast, the Ensemble ML model consistently registered the weakest performance, exhibiting FDRs in the double digits, peaking at 16.784% for the Normal class. The CAtt-BiLSTM model represented the best-performing baseline, but its FDR values (ranging from 1.671% to 4.783%) remained significantly higher than the near-zero rates achieved by the proposed method. This clear and consistent gap visually and numerically substantiated the profound superiority of the ZCAtt-BiLSTM model in achieving highly reliable and trustworthy positive attack predictions.

**Table 7:** Simulation analysis of the FDR metric across different methods.

Classes	CAtt BiLSTM	BiLSTM	CAE	BiGRU	Ensemble ML	Proposed (ZCAtt-BiLSTM)
Blackhole Attack (BA)	1.671%	2.676%	8.673%	5.872%	11.673%	0.0987%
Flooding Attack (FA)	1.843%	4.832%	10.743%	8.453%	14.784%	0.987%
Version Number Attack	3.745%	4.893%	9.563%	6.673%	12.674%	0.825%
Rank Attack	3.845%	5.893%	11.783%	8.437%	13.562%	0.7173%
Normal	4.783%	6.652%	12.674%	8.562%	16.784%	0.4372%

**Fig 19:**
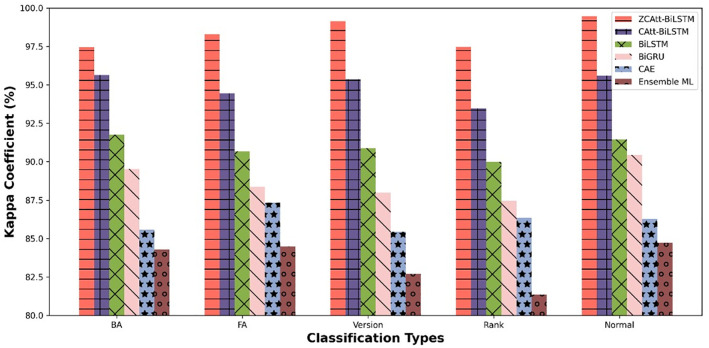
KC analysis for varying existing scheme.

Table 8 and Fig 19 presented a class-wise comparison of the Kappa Coefficient (KC) metric, which evaluated classification reliability by adjusting for agreement that occurred purely by chance. The analysis evaluated the Proposed (ZCAtt-BiLSTM) model against five baselines across the five attack and normal classes. The Proposed (ZCAtt-BiLSTM) model decisively demonstrated the highest KC score in every category, signifying its superior and non-random predictive strength across all attack types. Specifically, the proposed model achieved its highest KC score on the Normal class (99.467%), followed closely by the Version number attack (99.141%) and Flooding attack (98.291%). In contrast, the baseline models consistently showed significantly lower KC values, highlighting their greater reliance on chance agreement. The Ensemble ML model registered the lowest KC values, with its performance ranging from 81.345% (on Rank attack) to 84.725% (on Normal). This considerable numerical gap visually and numerically substantiated the profound and reliable superiority of the proposed ZCAtt-BiLSTM model in classifying specific RPL-IoT network attacks.

**Table 8:** Simulation analysis of the KC metric across different methods.

Classes	CAtt BiLSTM	BiLSTM	CAE	BiGRU	Ensemble ML	Proposed (ZCAtt-BiLSTM)
Blackhole Attack (BA)	95.641%	91.762%	85.578%	89.519%	84.294%	97.453%
Flooding Attack (FA)	94.442%	90.663%	87.331%	88.365%	84.483%	98.291%
Version Number Attack	95.367%	90.884%	85.452%	87.992%	82.692%	99.141%
Rank Attack	93.457%	89.986%	86.345%	87.456%	81.345%	97.472%
Normal	95.599%	91.448%	86.296%	90.439%	84.725%	99.467%

**Fig 20:**
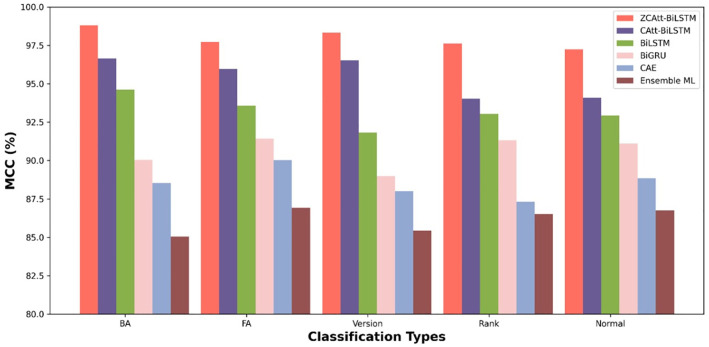
MCC analysis for varying existing schemes.

Table 9 and Fig 20 present a class-wise comparison of the Matthews Correlation Coefficient (MCC) metric, which evaluates the quality of binary and multiclass classifications by considering all four confusion matrix values. As the MCC provided a balanced measure, reliable even for imbalanced datasets, it served as a robust indicator of model performance. The analysis evaluated the Proposed (ZCAtt-BiLSTM) model against five baselines across the five attack and normal classes. The Proposed (ZCAtt-BiLSTM) model decisively demonstrated the highest MCC score in every category, signifying its superior and balanced predictive strength across all attack types. Specifically, the proposed model achieved its peak MCC score on the Blockhole attack (98.814%), followed by the Version number attack (98.341%) and Flooding attack (97.731%). In contrast, the baseline models consistently registered significantly lower MCC values, indicating a notable performance gap. The Ensemble ML model recorded the weakest performance, with its MCC values ranging from 85.052% to 86.745%. This considerable numerical and visual separation between the proposed model and all baselines visually and numerically substantiated the comprehensive, reliable superiority of the ZCAtt-BiLSTM architecture for multiclass cyberattack detection in RPL-IoT networks

**Table 9:** Simulation analysis of the MCC metric across different methods.

Classes	CAtt BiLSTM	BiLSTM	CAE	BiGRU	Ensemble ML	Proposed (ZCAtt-BiLSTM)
Blackhole Attack (BA)	96.656%	94.611%	88.528%	94.611%	85.052%	98.814%
Flooding Attack (FA)	95.952%	93.582%	90.021%	93.582%	86.922%	97.731%
Version Number Attack	96.522%	91.833%	88.002%	91.833%	85.442%	98.341%
Rank Attack	94.029%	93.044%	87.307%	93.044%	86.521%	97.621%
Normal	94.099%	92.948%	88.846%	92.948%	86.745%	97.238%

**Fig 21:**
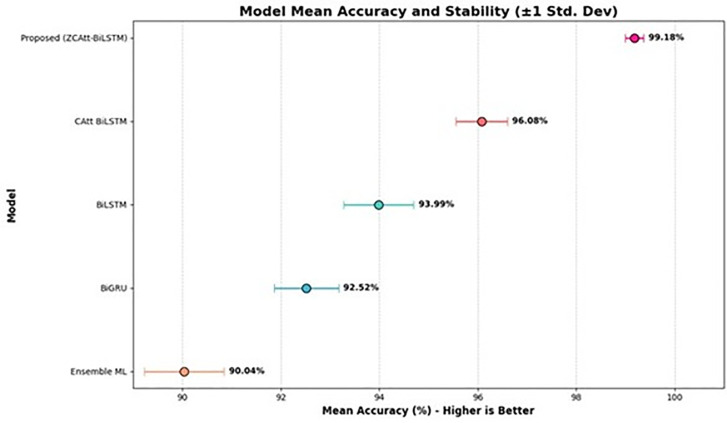
Statistical test visualization analysis across different frameworks.

Table 10 and Fig 21 presented a statistical test analysis to formally validate the significance of the performance differences between the proposed ZCAtt-BiLSTM framework and the five baseline models. The Mean ± Standard Deviation values showed that the proposed model achieved the highest mean accuracy of 99.18 pm 0.19, while the Ensemble ML model registered the lowest performance at 90.04 pm 0.81. Crucially, the statistical tests demonstrated the superiority of the proposed framework. The Wilcoxon test values for all baselines fell significantly below the conventional alpha = 0.05 threshold (e.g., 0.0032 for CAtt-BiLSTM, 0.0021 for BiLSTM), indicating that the performance difference between the proposed model and all baselines was statistically significant and not due to random chance. Similarly, the Paired t-test values, particularly the very low p-value of 0.0045 associated with the proposed model, substantiated its superior performance and reliability compared to the comparison models, thereby offering strong statistical proof of the framework’s effectiveness.

**Table 10:** Statistical test analysis across different frameworks.

Models used	Mean ± Std	Paired t-test	Wilcoxon test
**ZCAtt-BiLSTM (Proposed)**	99.18 ± 0.19	0.0045	0.0019
**CAtt BiLSTM**	96.08 ± 0.52	4.88	0.0032
**BiLSTM**	93.99 ± 0.71	5.74	0.0021
**BiGRU**	92.52 ± 0.65	2.15	0.0047
**Ensemble ML**	90.04 ± 0.81	1.39	0.0038

## Discussion

The experimental results demonstrate that the performance improvements are closely aligned with the methodological design, particularly the integration of Pareto scaling, Ch-PKO-based feature selection, and the ZCAtt-BiLSTM classification model. Each component of the framework contributes directly to achieving the objectives of improving detection accuracy, reducing computational overhead, and ensuring reliable multiclass classification in RPL-IoT networks. The preprocessing stage, which incorporates Pareto scaling, plays a key role in stabilizing feature distributions and improving model learning. By preserving variance while normalizing feature magnitudes, this step enables better separation between normal and malicious traffic patterns. The effectiveness of this stage is reflected in the consistent performance across evaluation metrics and the stable convergence behavior observed during training. The Ch-PKO-based feature selection mechanism further strengthens the framework by reducing data dimensionality while retaining the most informative attributes. The convergence analysis (Figs 12 and 13) shows that the algorithm rapidly stabilizes within approximately 15 iterations, indicating efficient optimization. The feature importance results (Fig 4) reveal that protocol-level and temporal features, such as *from*, *to*, *frame_proto*, and *lifetime*, are highly discriminative for attack detection. This targeted feature reduction minimizes redundancy and directly supports the objective of lowering computational overhead, making the approach more suitable for resource-constrained environments. The ZCAtt-BiLSTM model is the primary contributor to classification performance. The bidirectional LSTM component captures temporal dependencies in network traffic by processing sequences in both forward and backward directions, improving contextual understanding of attack patterns. The integration of the zero-parameter channel attention mechanism enhances feature relevance without increasing model complexity. This design choice is reflected in the achieved performance, including 99.37% accuracy and 98.57% F1-score (Table 2), along with strong precision and recall values. Comparative analysis with baseline models further validates the effectiveness of the framework. The improvement over the CAtt-BiLSTM model (Fig 6) indicates that the zero-parameter attention mechanism contributes to improved feature focus and reduced overfitting. This is supported by the learning curves (Figs 14 and 15), where training and validation metrics closely align, demonstrating stable convergence and good generalization capability. Class-wise evaluation provides additional evidence of robustness. The confusion matrix (Fig 11) shows accurate classification across all attack categories, with minimal misclassification compared to baseline approaches. The error rate analysis (Table 7) highlights a very low False Discovery Rate, indicating reliable detection with minimal false alarms. This directly supports the objective of achieving dependable intrusion detection in security-sensitive IoT environments. Furthermore, statistical validation (Table 10 and Fig 21) confirms that the observed performance improvements are significant and not due to random variation. While the results demonstrate strong performance, certain trade-offs are observed. The training time analysis (Fig 16) indicates increased computational cost due to the optimization process and deep learning architecture. Although this impacts efficiency, the improvement in detection accuracy, reliability (MCC/Kappa), and error reduction justifies this trade-off within the context of security-critical applications.

## Conclusion

This work presents a DL-based framework for multiclass RPL attack detection in IoT networks, combining Ch-PKO-based feature selection with a ZCAtt-BiLSTM classification model. The feature selection stage effectively reduces redundant attributes, improving computational efficiency and supporting faster convergence. The ZCAtt-BiLSTM model enhances classification performance by capturing temporal dependencies and improving feature discrimination through a zero-parameter attention mechanism. Experimental results demonstrate strong performance, achieving 99.305% accuracy, 98.57% F1-score, and 97.949% MCC, along with a low false discovery rate. Comparative evaluation shows consistent improvement over baseline models across multiple metrics. These findings indicate that the integration of optimized feature selection and attention-based sequence modeling improves detection accuracy and reliability in RPL-based IoT environments. However, the framework is evaluated using simulated datasets, which may not fully represent real-world network conditions. In addition, the training process introduces higher computational cost, which may impact scalability in highly dynamic environments. Future work will focus on validating the framework in real-world IoT deployments and exploring distributed learning approaches, such as federated learning, to improve scalability and adaptability.
